# COVID-19 and Good’s Syndrome After Thymectomy

**DOI:** 10.7759/cureus.70274

**Published:** 2024-09-26

**Authors:** Ingyin May, Srikanth Akunuri

**Affiliations:** 1 General Medicine, King's College Hospital NHS Foundation Trust, London, GBR

**Keywords:** covid-19, good's syndrome, immunodeficiency, recurrent infection, thymectomy

## Abstract

Good’s syndrome is a rare secondary immunodeficiency characterised by recurrent infection and hypogammaglobulinemia associated with thymus tumour, and symptoms of recurrent infection may persist even after thymectomy. It is characterised by increased susceptibility to bacterial, viral, and fungal infections, as well as autoimmunity. Immunoglobulin replacement therapy has been reported to decrease infection rate, hospitalization, and the rate of antibiotic use in these patients. There are very few reported cases of patients with COVID-19 infection and Good's syndrome; however, there is no strong association proven between these two situations. The patient we report in this article had a COVID-19 infection first, was incidentally diagnosed with thymoma, and got the symptoms of Good's syndrome after thymectomy.

## Introduction

Good’s syndrome is a rare adult-onset immunodeficiency first reported by Dr. Good in 1954 [1]. It is usually presented with thymoma, recurrent infection, and hypogammaglobulinemia. The clinical symptoms of recurrent infection can present before or after thymectomy. Thymoma is the most common neoplasm arising from the thymus and consists of neoplastic thymic epithelial cells and non-neoplastic maturing thymocytes. The incidence is 0.15 cases per 100,000 population per year, and the affected patients are usually aged 40-60 years. The pathogenesis of Good's syndrome is still unclear for many years. Although other autoimmune diseases like myasthenia gravis are common after thymectomy, Good's syndrome is a very rare presentation associated with thymoma or thymectomy. There are very few cases reported for patients with Good's syndrome and COVID-19 infection, but there is no strong proof of association from an immunological perspective. During the last three years, there have been lots of incidences of COVID-19 in patients with primary or secondary immunodeficiency. However, there is no strong evidence of an association between COVID-19 and thymoma or Good’s syndrome. In this report, we present a case of a middle-aged man who was diagnosed with thymoma after a COVID-19 infection, followed by a recurrent systemic infection, which is Good’s syndrome after thymectomy [2-4].

## Case presentation

**Figure 1:**
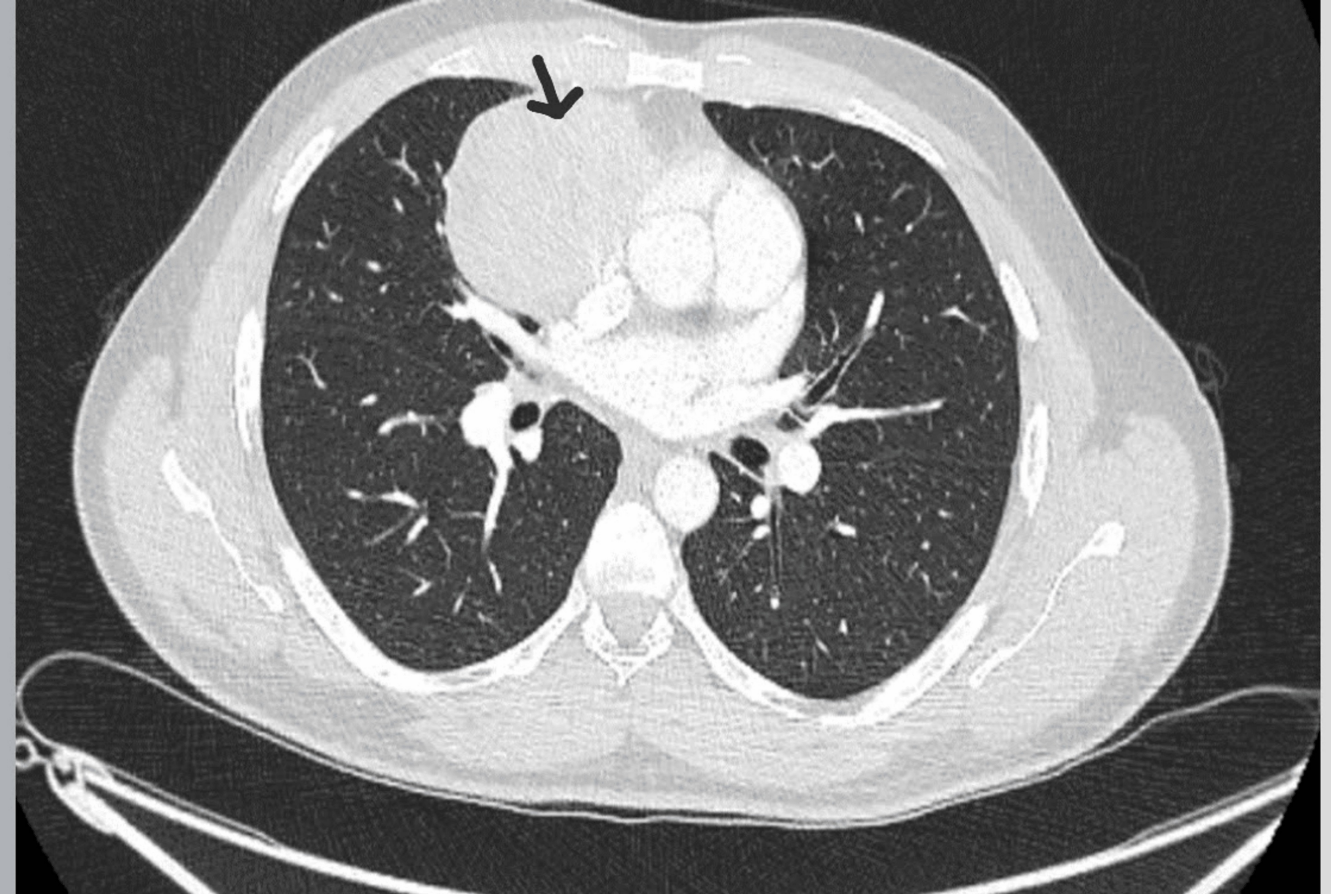
Chest CT conducted in 2022 showing a large anterior mediastinal mass.

A gentleman in his 30s was admitted to our hospital with COVID-19 infection in 2022 when a 7.5 cm large soft tissue mass was incidentally found on his chest CT (Figure 1). The mass was confirmed to be T1a N0 M0 type AB thymoma by PET scan and ultrasound-guided tissue biopsy. He had thymectomy surgery in late 2022, which was complicated by hospital-acquired pneumonia and he was treated with a course of intravenous tazobactam and piperacillin 4.5 grams three times a day at that time.

**Figure 2:**
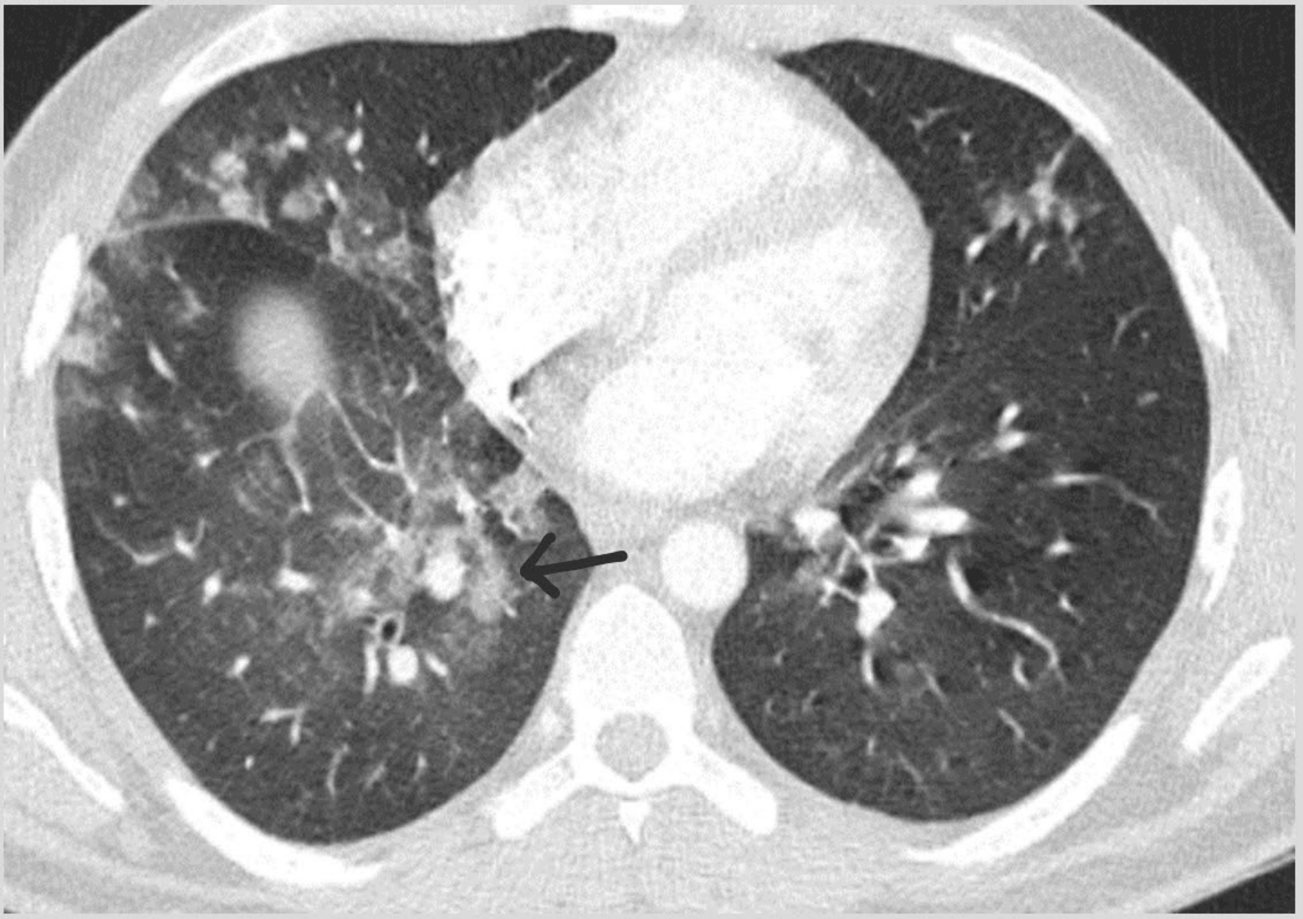
Chest CT conducted in 2023 showing infective changes in lung tissues.

A few months after surgery, the patient repeatedly went to his general practitioner and emergency department multiple times due to purulent cough, fever, generalised discomfort, vomiting, and diarrhoea. In August 2023, he was heavily investigated by the respiratory team, and he had an MRI of the thorax and another CT of the chest, which showed infective changes and malignancy was excluded (Figure 2). The sputum culture and bronchoscopy results were also negative for tuberculosis. The repeated PET scan revealed no residual thymoma. His HIV test was negative, and he did not have a past medical history of diabetes, bronchiectasis, or other immunodeficient problems previously.

Our patient was reported to have lost weight because of a recurrent infection. There was no change in his bowel habits, and no palpable lymph nodes were found all over his body. He did not report to have any blood in the stool as well. He was a non-smoker and occasionally drank alcohol.

During another hospital admission in early 2024 with a similar problem, an immunology work-up was done, which showed low immunoglobulin levels: IgA = 0.14, total IgG = 2.84, IgM < 0.05, serum kappa light chains = 2.93, and serum lambda light chains = 3.08 (Table 1). The sputum culture was positive with heavy growth of *Haemophilus influenzae* during that admission; however, he tested negative for COVID-19 and influenza virus. The connective tissue screening and autoimmune screening were also negative. The physical examination was reported to have occasional crackles over the middle zone of the left side of the chest, but no other significant findings were found. He was responsive to a course of antibiotic therapy.

**Table 1:** Core immunology level in mid-2024. IgA: immunoglobulin A; IgG: immunoglobulin G; IgM: immunoglobulin M; IgE: immunoglobulin E.

Core immunology level	Reference range & units	Result
IgA	0.8-3.0 g/L	0.14
Total IgG	6.0-16.0 g/L	2.84
IgM	0.4-2.5 g/L	<0.05
IgE	0.0-81.0 kU/L	<2

He was followed up by a rapid diagnosis clinic in mid-2024 and the repeated immunoglobulin test showed IgG1 at 1.64, IgG2 at 0.59, IgG3 at 0.13, and IgG4 at 0.13, which were still lower than normal range and got referred to immunology department (Table 2). While he was waiting for an immunology appointment, he had another visit to the emergency department a few weeks later and was commenced on a course of oral antibiotics.

**Table 2:** Immunoglobulin level after two months. IgG: immunoglobulin G.

Immunoglobulin subclasses	Reference range & units	Result
IgG 1 concentration	3.82 - 9.29 g/L	1.64
IgG 2 concentration	2.42 - 7.00 g/L	0.59
IgG 3 concentration	0.22 - 1.76 g/L	0.13
IgG 4 concentration	0.04 - 0.86 g/L	0.13

## Discussion

Good's syndrome is a condition in which thymoma is associated with hypogammaglobulinemia. Thymoma resection does not resolve hypogammaglobulinemia or the associated immunodeficiency, which can lead to persistent susceptibility to infections. Patients with thymoma frequently present with autoimmune disorders, mostly myasthenia gravis and very rarely Good’s syndrome, in which hypogammaglobulinaemia and recurrent bacterial infection persist even after thymus surgery. The incidence rate of Good's syndrome is estimated to be 1.5 per 100,000 [4]. Due to the variable clinical presentation of Good's syndrome, the diagnosis is hard to recognise to get an early diagnosis and treatment. The main difference between Good's syndrome and common variable immunodeficiency (CVID) is that Good's syndrome is an adult-onset acquired type mainly affecting B-cells and immunoglobulins while CVID is a congenital immunodeficiency affecting both B cells and T cells since birth or young age. In Good's syndrome, symptoms usually start only after thymectomy or before thymus resection. It is still challenging to explain in detail about Good's syndrome due to its unclear pathogenesis for many years.

In a study by Zaman et al., the most common histological type was AB [5]. In contrast to the patients with CVID, those with Good's syndrome have very low levels of B cells and immunoglobulins. It is also reported to have no improvement in B-cell and immunoglobulin levels after thymectomy [5]. Some patients present with recurrent infections after thymectomy. The prognosis of Good's syndrome is reported to be poorer compared with other immunodeficiencies [1].

In our patient, we found his thymoma incidentally after he was hospitalised with COVID-19 infection and subsequently got recurrent infection after the surgery. He had no previous history of similar problems before he had the surgery. The diagnosis of Good’s syndrome is reported to be still under-appreciated. Although there are some possibilities associated between COVID-19 infection and Good's syndrome from the immunological perspective, the number of cases is very few to prove. Therefore, further case reports and more information about these two conditions are needed to correlate the clinical conditions. Our patient presented with COVID-19 infection first before we found out his thymoma, hence the immunoglobulin levels were not tested in the first place. The mainstay treatment is the regular administration of immunoglobulin replacement to prevent recurrent infection and supportive treatment including antibiotics and regular immunology follow-up.

## Conclusions

To conclude, this patient was referred to the immunology clinic for further diagnosis, investigation, and treatment. This case report highlights to be aware of Good’s syndrome in post-thymectomy patients with recurrent infections and to do immunology work up with immunoglobulin levels in those patients. Timely replacement of immunoglobulin is reported to reduce recurrent infections. Therefore, we are hoping that this case report will be helpful for clinicians to get an early accurate diagnosis and effective treatment plan for patients with susceptible recurrent infections after thymectomy.
